# Systems Biology Methods Applied to Blood and Tissue for a Comprehensive Analysis of Immune Response to Hepatitis B Vaccine in Adults

**DOI:** 10.3389/fimmu.2020.580373

**Published:** 2020-11-04

**Authors:** Rym Ben-Othman, Bing Cai, Aaron C. Liu, Natallia Varankovich, Daniel He, Travis M. Blimkie, Amy H. Lee, Erin E. Gill, Mark Novotny, Brian Aevermann, Sibyl Drissler, Casey P. Shannon, Sarah McCann, Kim Marty, Gordean Bjornson, Rachel D. Edgar, David Tse Shen Lin, Nicole Gladish, Julia Maclsaac, Nelly Amenyogbe, Queenie Chan, Alba Llibre, Joyce Collin, Elise Landais, Khoa Le, Samantha M. Reiss, Wayne C. Koff, Colin Havenar-Daughton, Manraj Heran, Bippan Sangha, David Walt, Mel Krajden, Shane Crotty, Devin Sok, Bryan Briney, Dennis R. Burton, Darragh Duffy, Leonard J. Foster, William W. Mohn, Michael S. Kobor, Scott J. Tebbutt, Ryan R. Brinkman, Richard H. Scheuermann, Robert E. W. Hancock, Tobias R. Kollmann, Manish Sadarangani

**Affiliations:** ^1^ Vaccine Evaluation Center, BC Children’s Hospital Research Institute, Vancouver, BC, Canada; ^2^ Telethon Kids Institute, University of Western Australia, Nedlands, WA, Australia; ^3^ Centre for Microbial Diseases and Immunity Research, University of British Columbia, Vancouver, BC, Canada; ^4^ Simon Fraser University, Burnaby, BC, Canada; ^5^ Department of Informatics, J. Craig Venter Institute (La Jolla), La Jolla, CA, United States; ^6^ Terry Fox Laboratory, Vancouver, BC, Canada; ^7^ Prevention of Organ Failure (PROOF) Centre of Excellence and Centre for Heart Lung Innovation, St. Paul's Hospital, Vancouver, BC, Canada; ^8^ Centre for Molecular Medicine and Therapeutics, Department of Medical Genetics, University of British Columbia, Vancouver, BC, Canada; ^9^ Department of Biochemistry and Molecular Biology, Faculty of Medicine, University of British Columbia, Vancouver, BC, Canada; ^10^ Translational Immunology Lab, Institut Pasteur, Paris, France; ^11^ Department of Immunology and Microbiology, The Scripps Research Institute, La Jolla, CA, United States; ^12^ IAVI Neutralizing Antibody Center, The Scripps Research Institute, La Jolla, CA, United States; ^13^ Center for Infectious Disease and Vaccine Research, La Jolla Institute for Immunology (LJI), La Jolla, CA, United States; ^14^ Human Vaccines Project, New York, NY, United States; ^15^ Department of Radiology, BC Children’s Hospital, Vancouver, BC, Canada; ^16^ Wyss Institute at Harvard University, Department of Pathology, Brigham and Women’s Hospital, Harvard Medical School, Boston, MA, United States; ^17^ British Columbia Centre for Disease Control, Vancouver, BC, Canada; ^18^ Department of Microbiology and Immunology, Life Sciences Institute, University of British Columbia, Vancouver, BC, Canada; ^19^ Department of Medicine, Division of Respiratory Medicine, University of British Columbia, Vancouver, BC, Canada; ^20^ Department of Medical Genetics, University of British Columbia, Vancouver, BC, Canada

**Keywords:** multi-omic, single cell, lymph node, gene expression, bio-informatic, immunimonitoring, vaccine

## Abstract

Conventional vaccine design has been based on trial-and-error approaches, which have been generally successful. However, there have been some major failures in vaccine development and we still do not have highly effective licensed vaccines for tuberculosis, HIV, respiratory syncytial virus, and other major infections of global significance. Approaches at rational vaccine design have been limited by our understanding of the immune response to vaccination at the molecular level. Tools now exist to undertake in-depth analysis using systems biology approaches, but to be fully realized, studies are required in humans with intensive blood and tissue sampling. Methods that support this intensive sampling need to be developed and validated as feasible. To this end, we describe here a detailed approach that was applied in a study of 15 healthy adults, who were immunized with hepatitis B vaccine. Sampling included ~350 mL of blood, 12 microbiome samples, and lymph node fine needle aspirates obtained over a ~7-month period, enabling comprehensive analysis of the immune response at the molecular level, including single cell and tissue sample analysis. Samples were collected for analysis of immune phenotyping, whole blood and single cell gene expression, proteomics, lipidomics, epigenetics, whole blood response to key immune stimuli, cytokine responses, *in vitro* T cell responses, antibody repertoire analysis and the microbiome. Data integration was undertaken using different approaches—NetworkAnalyst and DIABLO. Our results demonstrate that such intensive sampling studies are feasible in healthy adults, and data integration tools exist to analyze the vast amount of data generated from a multi-omics systems biology approach. This will provide the basis for a better understanding of vaccine-induced immunity and accelerate future rational vaccine design.

## Introduction

Human vaccination is one of the greatest achievements in medical history. However, failure to develop highly effective vaccines for tuberculosis, HIV, malaria, or cancers emphasizes that the traditional trial-and-error approach is limited, and our incomplete understanding of how vaccines work does not yet enable successful rational vaccine design (1). An ideal vaccine would provide significant protection for a long (lifelong) time following only a single dose (1, 2). Few current vaccines reach this ideal, but some successes show that it is possible. New technologies including multi-omic systems biology offer powerful solutions but its potential has not yet been fully realized.

One of the main reasons for the lack of insight into the mechanisms underlying vaccine-induced protection is that human clinical studies have typically studied the response to vaccines in blood only, collected over few time points after vaccination. While blood sampling coupled with systems biology is a practical and a potentially informative proxy for the tissue-resident host response to vaccination, it likely does not capture the full range of cellular and molecular interactions in the tissue, most importantly the draining lymph node (LN), which is the site of the primary immune response to vaccination. This is due to practical limitations in obtaining relevant tissue samples in humans, necessitating assumptions based on animal, *in vitro* and/or *ex vivo* models (3–5). A complete understanding of human immune responses to vaccination will only be possible with collection of tissue samples, in addition to blood, during clinical trials.

The aim of this study was to demonstrate feasibility of a comprehensive blood- and tissue-based systems biology vaccine study in humans, establish the necessary infrastructure, and undertake analysis of pre- and post-vaccine samples to identify markers of a protective immune response. In this article we will describe in detail the methods utilized in the study for sample collection, sample processing and data analysis. A set of the data generated are included in the companion manuscript (“Multi-omic data integration allows baseline immune signatures to predict hepatitis B vaccine response in a small cohort”) and were used for multi-omic data integration to identify baseline signatures that can predict vaccine response.

## Methods

### Study Locations and Participants

**Figure 1:**
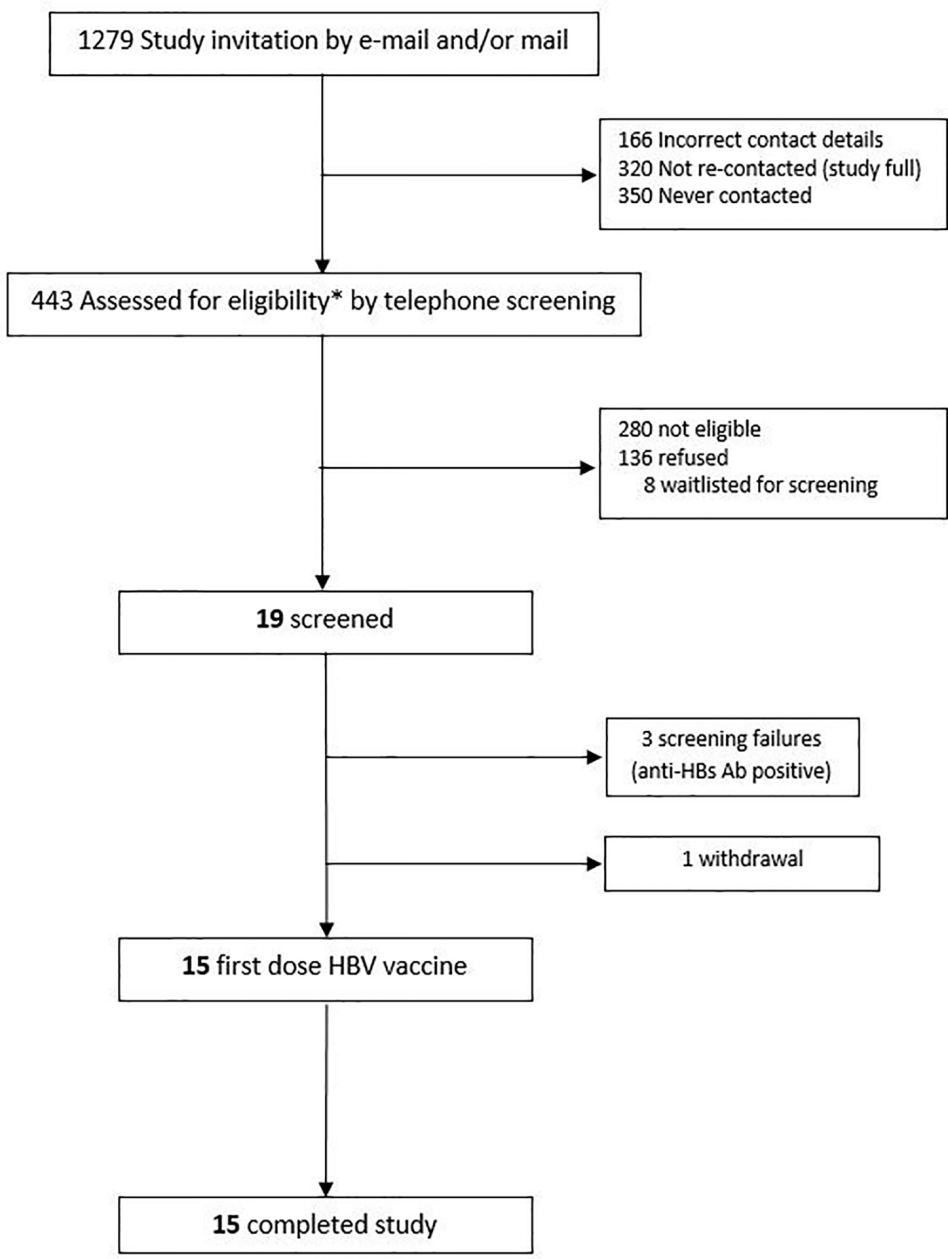
Study participants recruitment strategy.

This was a prospective, observational study (ClinicalTrials.gov; NCT03083158) of immune responses to hepatitis B virus (HBV) vaccine, with recruitment occurring at the Vaccine Evaluation Center (VEC), BC Children’s Hospital, Vancouver, Canada in accordance with a local ethics committee-approved protocol (Ethics Ref: H17-00175). All initial sample processing was undertaken at the VEC laboratory. Other laboratory work occurred at various institutions in Vancouver, La Jolla, Boston, and Paris. Participants were healthy adults, aged 40–80 years, who were seronegative to HBV at the time of enrolment. Eligibility criteria are shown in **Table 1**. Participants were recruited by e-mail, mail and telephone *via* an existing “permission to contact for research” database held at the VEC (**Figure 1**).

**Table 1:** Study inclusion and exclusion criteria.

Inclusion criteria	Exclusion criteria
• Healthy adult aged 40–80 years • No history of hepatitis B disease • No prior receipt of any hepatitis B-containing vaccine • Undetectable level of anti-HBs and anti-HBV core (HBc) antibody and HBs antigen at study enrolment • Generally good health (stable chronic conditions acceptable) • Living independently or with minimal assistance (clinical frailty score 1–5)^78^ and able to attend all study visits • Willing and able to comply with the requirements of the protocol • Provided informed consent for participation in the study	• Individual on the study delegation log • History of being a household contact of a known hepatitis B-infected individual • Planned administration of any vaccine not specified in the study protocol from 1 month pre- to the 1 month post-1^st^ dose of vaccine • Planned receipt of any investigational drug during the study • Confirmed or suspected immunodeficiency • Family history of congenital or hereditary immunodeficiency • Receipt of >1 week of systemic immunosuppressants or immune modifying drugs in the 3 months prior to dose 1 of vaccine • Taking any anti-platelet or anti-coagulant medications (excluding daily low-dose aspirin) • Bleeding disorder or thrombocytopenia, that contraindicates intramuscular injection, blood collection and/or lymph node fine needle aspiration • Administration of immunoglobulins within the prior 12 months and/or any other blood products within the prior 3 months or planned during the study period • Current pregnancy or planning to become pregnant in the 6 months post-dose 1 vaccination • History of allergy to any component of the vaccine • Unstable medical condition, as indicated by a requirement for hospitalization or a substantial medication change to stabilize said condition within previous 3 months • History of any neurologic disorders or seizures, including a history of Guillain-Barré syndrome • Clinical Frailty score of 6–7 (moderately frail or severely frail)^78^ • Scheduled elective surgery or other procedures requiring general anaesthesia from 1 month pre- to the 1 month post-1^st^ dose of vaccine • Any other significant disease or disorder which, in the opinion of the Investigator, may either put the participants at risk because of participation in the study, or may influence the result of the study, or the participant’s ability to participate in the study.

### Biospecimen Collection

**Figure 2:**
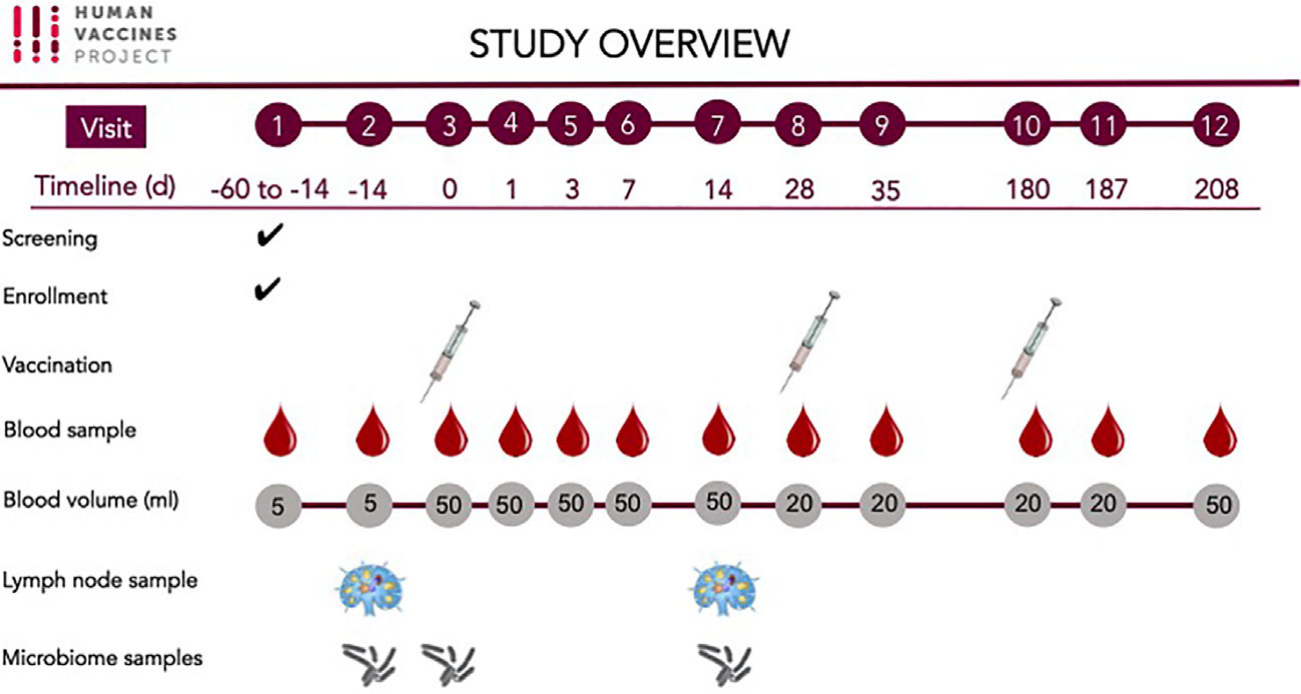
Study Overview.

Peripheral blood samples, LN aspirates and microbiome samples were obtained for the investigation of host responses associated with HBV vaccine (**Table 2** and **Figure 2**).

**Table 2:** Sample collection details per omic assay and study visits.

	Study Visits											
	V1	V2	V3	V4	V5	V6	V7	V8	V9	V10	V11	V12
Assay or Sample Type												
Complete blood count	**√**	**√**	**√**	**√**	**√**	**√**	**√**	**√**	**√**	**√**	**√**	**√**
Hepatitis B Serology	**√**							**√**		**√**		**√**
CMV Serology			**√**									
Lymph node biopsy		**√**					**√**					
Microbiome samples		**√**	**√**				**√**					
Whole blood RNA-seq			**√**	**√**	**√**	**√**	**√**					
Epigenetics			**√**	**√**	**√**	**√**	**√**					
Proteomics & lipidomics			**√**	**√**	**√**	**√**	**√**					
Immune phenotyping			**√**	**√**	**√**	**√**	**√**					
Cell mediated Immunity		**√**						**√**		**√**		**√**
Single cell RNA-seq			**√**	**√**	**√**	**√**	**√**					
Milieu interieur			**√**									
Antibody repertoire			**√**									
Cytokine Expression			**√**				**√**			**√**		**√**

### Blood Sample Collection

Peripheral blood was obtained from an easily accessible vein (e.g., back of the hand and elbow crease) by experienced staff at the study site. Blood was drawn in three distinct collection tubes, each one appropriate for the downstream protocol. A large bore butterfly needle (19-21G) was used and the collection tubes inserted into the device as follows: an EDTA-treated vacutainer tube (BD, cat #366643) for complete blood counts (3 ml) then an IVD PAXgene blood collection tube (BD, Cat. No. 762165) for RNA samples (2.5 ml) and finally a pre- heparinized 60-ml syringe for the rest of the samples (~50 ml).

### Lymph Node Aspirate Collection

Cells from the vaccine-draining axillary LN were obtained *via* fine needle aspiration (FNA) conducted by an interventional radiologist at the study site. A 22-gauge needle attached to a 5- to 10-ml syringe was passed into the LN *via* ultrasound guidance. The needle was moved back and forth within the LN for 30 s under negative pressure, whereby a 2-ml suction was applied by pulling back the syringe plunger. The needle was then withdrawn, and the contents were immediately expelled into 5 ml of cold sterile complete medium containing 2 mM L-glutamine (RPMI, Gibco cat #72400-047). The needle was rinsed in cold medium 3 to 4 times to collect as many cells as possible. The cells were then placed on ice and immediately transported to the laboratory for further processing.

### Microbiome Sample Collection

Microbiome samples were obtained prior and post vaccination using EZ II culture swabs (BD, cat #220145) for fecal samples and EZ I culture swabs (BD cat #220144) for nasal, oral and skin samples. The microbiome samples were chosen to be taken at those two time points to address two aspects: whether the vaccine will change the microbiome and whether the microbiome a person has prior to vaccination correlates with the individual’s response to vaccination. All swabs except the fecal swabs were collected by study staff at the study visits.

#### Fecal Sample Collection

Fecal samples were collected by the study participants at home the day before their study visit. The participants were given a collection kit containing a pair of gloves, a sealed swab, a biohazard bag for transport, and clearly illustrated instructions detailing the collection process. The amount of biomass required was small, saturating only half of the swab to allow optimal downstream processing. The biohazard bags with the fecal samples were kept at 4°C in the participant’s refrigerator and brought to the clinic visit by study participants within 20 h of collection. Upon arrival to the clinic, the swabs were transferred to the lab and saved without further processing at −80°C for microbiome analysis.

#### Skin Samples Collection

The participants were asked not to wash their faces for at least 2 h prior to sample collection. Prior to sampling, a sterile swab was moistened in an SCF-1 vial. Wearing gloves, the study staff stretched the skin site taut with one hand and with the other hand held the swab so the shaft was parallel to the skin surface and rubbed the swab over the skin surface. Both sides of the cotton tips were firmly rubbed back and forth about 50 times over the skin surface by applying firm pressure. The swab was then sealed and transported to the laboratory to be stored at −80°C until microbiome analysis.

#### Nasal Sample Collection

The nasal samples were collected using a sterile fresh, dry swab. The swab was inserted approximately 0.5 cm into the nasal cavity. The inside of both nares were swabbed by rubbing the swab along the walls of the nares 5 time. The collection tube was then sealed, place in a biohazard bag and transported within 4 h of collection to the laboratory where it was stored at −80°C until further microbiome analysis.

#### Oral Sample Collection

The oral swabs were collected using a fresh sterile dry swab. Both sides of the cotton tips were firmly rubbed on the surface of the tongue without touching the inside of the cheeks, teeth or lips. The collection tube was then sealed and placed in a biohazard bag before being transported to the lab and stored within 4 h at −80°C for future microbiome analysis

## Blood and Lymph Node Sample Pre-Processing for Multi-Omic Analysis

### Lymph Node Cell Processing and Cryopreservation

Freshly collected LN cells were separated by centrifugation at 450 × *g* for 5 min in a pre-cooled centrifuge and resuspended in cold 0.5 ml of heat inactivated fetal bovine serum (HIFBS) (Hyclone, cat #SH30071.0). If red blood cell contamination was observed, we planned to add 2 ml of ACK RBC lysis buffer (Thermo Fisher Gibco cat #A1049201) for 3 min to the cell suspension. To stop the reaction, 10 ml of phosphate buffer saline (PBS) with 2% HIFBS is to be added and the cells separated by centrifugation in the same conditions. In this particular cohort, due to a relatively small number of cells obtained, no RBC lysis was performed on the fine needle aspirate samples collected. The cells were then counted with a hemocytometer using a trypan blue dye, resuspended in a solution containing 90% FBS and 10% DMSO (Hybrimax, Sigma, cat #D2650) and transferred to a pre-labeled chilled cryovial. The LN aspirate samples were stored in liquid nitrogen for future flow cytometry analysis.

### Blood Sample Pre-Processing and Storage

**Figure 3:**
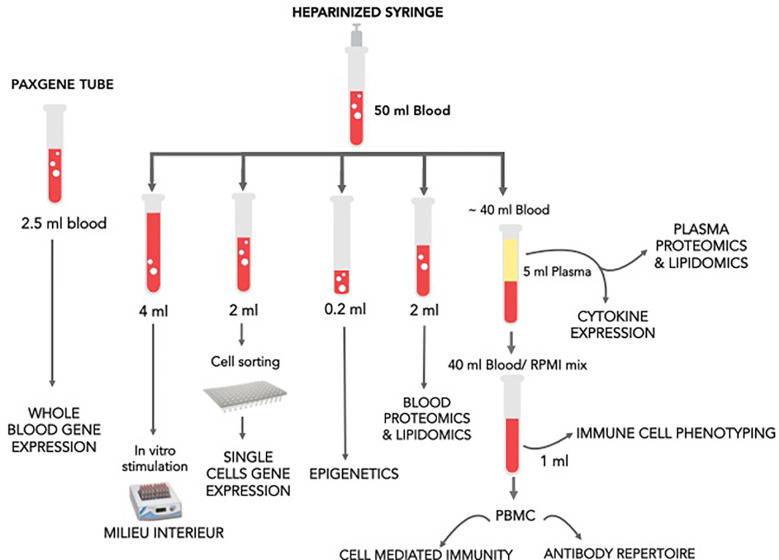
Omic sample processing overview.

Blood samples were processed as shown in **Figure 3**. The goal was to determine changes in cell composition, transcriptome (whole blood and single cell), white blood cell (WBC) and plasma proteome, metabolome, as well as WBC epigenome and next-generation sequencing (NGS) of VDJ regions in antigen-specific B cells clones obtained from peripheral blood mononuclear cells (PBMCs). Pre-vaccine samples were also assessed for the immune response to generic stimuli using Milieu Interieur’s TruCulture platform. RNA, proteins, and certain metabolites are susceptible to time and temperature dependent changes; thus, care was taken to process samples as fast as possible. The order of sample processing is dependent on the sample type sensitivity to degradation and changes over time. In this study, the samples were processed in order below.

#### Blood RNA Samples

Shortly after collection, the blood PAXgene tubes were inverted 10 times and kept at room temperature for a minimum of 2 h for complete lysis of blood cells. The tubes were then stored at −80°C for further transcriptomics analysis.

#### Single Cell RNA Samples

A total of 1.5 ml of blood sample was used to stain, single sort the cell populations of interest and perform subsequent single cell sequencing. Initially, 20 mM of EDTA (Fisher #BP120-500) was first diluted 10 times in the blood sample and red blood cells were lysed by adding RBC lysis buffer (eBiosciences, cat #00-4333-57) per manufacture’s recommendations. The mix was then resuspended with a sterile pipette and incubated at RT for 10 min, with gentle vortexing every 3 min. To stop the reaction, PBS was added, and the cell suspension separated by centrifugation at 500 **×** *g* for 5 min. The supernatant was then gently aspirated, and the red blood cell free suspension was resuspended in 3 ml of PBS. Before proceeding to cell staining, 100 µlS cells that served as a negative control for the flow cytometry sorting were set aside for machine calibration. The cells were then were separated by centrifugation at 600 **×** *g* for 10 min and the cell pellet resuspended in an antibody cocktail mixture (**Table 3**) diluted in PBS and 0.5% BSA (Bovine serum Albumine Sigma Aldrich, cat #A7906) according to the manufacture’s recommendations. A fixable viability dye (APC-eFluor 780 cat #65-0865-14) was also added to the cells prior to staining to allow sorting only viable cells. The cell mixture was incubated at RT protected for light for 30 min then washed once in PBS and resuspend in 3 ml of PBS for immediate sorting. The cell types of interest (neutrophils, plasmacytoid DCs, myeloid DCs, NK cells, and monocytes) were single sorted BD FACS Aria chilled chambers. The efficiency of single cell sorting was assessed by microscopy using fluorescent beads. Single cells were sorted into 96-well plates chilled on ice, pre-loaded with 2-μl lysis buffer (0.2% Triton X-100, (Sigma Aldrich, cat #9002-93-1), 2 Units/µl RNase inhibitor (Applied Biosystems, cat #N8080119), 1:2,000,000 dilution of ERCC spike-in RNAs (Life technologies, cat #4456740) and spun down at 300 **×** *g* at 4°C for 1 min before sorting. Negative and positive samples consisting of no cells per well and 100 cells per well were included in the plate design to use as controls in the Smart-seq library prep and sequencing. After sorting, each plate containing the single cell lysates was immediately sealed, placed on dry ice, and stored at −80°C.

**Table 3:** Antibody panel for cell sorting for single cell RNA sequencing.

Marker	Target	Fluorochrome	Clone	Catalog number
CD11c	Myeloid dendritic cells (mDC)	APC	Bu15	BD #340544
CD123	Plasmacytoid dendritic cells (pDC)	PE-Cy7	6H6	ebio #25-1239-42
CD3	T cell lineage	PE-CF-594	UCHT1	BD #562280
CD56	NK cells	PerCP Cy5	HCD56	BioLegend # 318343
CD11b	Mature and Immature Neutrophils	PE	ICRF44	Biolegend #301346
CD66	Neutrophils	PerCP Cy5	ASL-32	Biolegend #92719
CD16	Neutrophils, pro-inflammatory and transitional monocytes and NK cell subsets	FITC	3G8	Pharmingen #560996
HLA-DR	Dendritic cells, monocytes and B cells	eFlour605	LN3	ebio # 83-9956
CD14	Classical and intermediate monocytes	V500	M5E2	BD #561391
CD45	Pan leukocyte antigen	V450	HI30	BD #560367

#### Epigenetic Whole Blood Samples

Two hundred microliters of heparinized blood was transferred in a cryovial and saved at −80°C without any further processing to allow subsequent epigenetic analysis.

#### White Blood Cell Proteomic Samples

Two milliliters of 10× RBC lysis buffer (eBiosciences, cat #00-433-57) was diluted in 18 ml of ultrapure water and added to 2 ml heparinized whole blood to lyse red blood cells according to manufacturer’s instructions. Briefly, the cell suspension was incubated at room temperature for 15 min with gentle vortexing during the incubation time. Warm PBS was then added to mix to stop the reaction and the cells were separated by centrifugation at 600 **×** *g* for 10 min to collect the red blood cell free pellet. After an additional wash in PBS, the supernatant was carefully aspirated without disrupting the pellet and the WBC pellet was saved at −80°C to allow subsequent proteomic analysis.

#### Milieu Interieur Samples

The goal was to stimulate whole blood *in vitro* in a standardized stimulation system. Three core stimuli, plus a null control, were selected to best capture diverse immune responses: LPS; Poly(I:C) and SEB. One milliliter of heparinized whole blood was transferred into TruCulture tubes (with 2 ml media) within 15 min of blood collection, inserted into a dry block incubator, and maintained at 37°C ( ± 1°C) in room air for 22 h as previously described (6). At the end of the incubation period, tubes were opened, and a valve was inserted to separate the sedimented cells from the supernatant, stopping the stimulation reaction. Supernatants were saved for later Luminex testing at −80°C. For stabilization of RNA, 2 ml of Trizol LS (Sigma) was added to the cell pellet, vigorously vortexed and frozen at −80°C until analysis.

#### Plasma Samples

The remaining volume of heparinized blood (~30 ml) was placed in 50 ml conical tubes and underwent centrifugation at 1,000 **×** *g* for 10 min. The entire plasma component was then placed into new 50 ml conical tube and the mix inverted 4 times. The plasma was immediately aliquoted and saved at −80°C for subsequent analysis of the plasma proteome, lipidome and metabolome.

#### Immune Phenotyping Samples

The remaining cellular blood fraction left over after plasma collection was diluted twice in RPMI, mixed up and down and the cells/RPMI mixture was kept on wet ice. A total of 900 µl of the mix was stored for subsequent flow cytometry analysis. Whole blood cells were processed as previously described (7) using Smart tube lysing and fixing solutions as per manufacture’s recommendations. Briefly, 225 µl of diluted heparinized blood was added to four cryovials and mixed with 22 µl of 20 mM EDTA. The cell suspensions were mixed up and down with gentle pipetting, and 2 µl of fixable viability dye (eBioscience #65-0865-14) was added to each vial and the cells incubated for 30 min at 4°C protected from light. Red blood cells were then lysed by adding 350 µl of Smart lysis buffer (SmartTube Stable-Lyse V2) to each vial, incubated for 15 min at RT and the stained cells fixed with 1 ml Smart store buffer (SmartTube Stable-Store V2) for an additional 15 min. The fixed cells were then transferred to −80**°**C and stored until flow cytometry analysis were performed. Complete blood counts were undertaken shortly after blood draw from fresh samples by BC Children’s Hospital clinical laboratory to enumerate total WBC count, plus a differential count of neutrophils, lymphocytes and monocytes using a Sysmex XN-1000 Hematology Analyzer.

#### PBMC Samples

The remainder of the blood-RPMI mix was used for cryopreservation in order to allow T cell mediated immunity analysis by flow cytometry and NGS of VDJ regions in antigen-specific B cells. The blood was diluted twice in warm RPMI and the mixture gently flowed down the side of a tube, layered onto an identical volume of ficoll-paque (Amersham-Pharmacia, cat #17-1440-02) without breaking the surface plane. The tubes were then subjected to centrifugation at 900 **×** *g* for 20 min with no brake to allow the gradient to form. At the end of the centrifugation time, the cloudy interface containing the mononuclear cells were transferred to PBS and washed twice in the same medium. Every 10 million PBMCs were then resuspended in cold 0.5 ml of 12.5% human serum albumin (Sigma, cat #A5843) and 2× cold freezing buffer (90% HSA at 12.5%, 20 DMSO). The PBMC vials were saved at −80°C overnight in a freezing container (Mr Frosty Nalgene, cat #5100-000) and then transferred to liquid nitrogen until analysis.

## Multi-Omic Analysis

### Whole Blood Gene Expression

RNA extraction was performed on blood collected in PAXGene tubes using the PAXGene RNA purification kit (QIAGEN), following the manufacturer’s protocol. The Agilent 2100 Bioanalyzer (Santa Clara, CA, USA) was used to quantify and assess quality of total RNA. Poly-adenylated RNA was isolated using the NEBNext Poly(A) mRNA Magnetic Isolation Module (NEB, Ipswich, MA, USA). cDNA libraries were created from poly-adenylated RNA using the KAPA Stranded RNA-Seq library preparation kit (Roche, Basel, Switzerland). Samples were sequenced on the HiSeq2500 with single end reads of length 100 bp. Samples were checked for batch effects *via* heatmap analysis of normalized gene counts and none were found. Sequence quality of fastq files was assessed using FastQC v0.11.7 (https://www.bioinformatics.babraham.ac.uk/projects/fastqc/). Alignment of the raw reads to the reference genome was done using STAR v2.5.4b (8) to generate position-sorted BAM files. The genomic index required by STAR was created using the human genome downloaded from Ensembl (9), build GRCh38 v91, primary assembly. Gene counts were generated using the htseq-count function from HTSeq v0.10.0 (10). Results from the above programs (FastQC, STAR, and HTSeq) were compiled into a single report using MultiQC v1.0 (11). Analysis of RNA-Seq data was conducted using R v3.5.0 (https://www.R-project.org/) and RStudio v1.1.453 (http://www.rstudio.com/). Count files were filtered to remove genes with fewer than 10 counts across 15 samples (the number of biological replicates). Known globin genes were removed corresponding to the following six Ensembl IDs: ENSG00000206172, ENSG00000188536, ENSG00000244734, ENSG00000223609, ENSG00000213934, and ENSG00000196565. After filtering and globin removal, samples had a median library size of 4,842,497, with a minimum of 2,217,471 and maximum of 14,755,180. DESeq2 v1.20.0 (12) was used to identify differentially expressed (DE) genes. DE genes were identified using the Wald statistics test, followed by filtering for significance using a combined threshold of adjusted p-value ≤ 0.05 and absolute fold-changes ≥ 1.5. For pathway enrichment analysis, Sigora v2.0.1 (13) was used with the Reactome database at a hierarchy level of four. Correction for multiple comparisons was done using the Bonferroni method, filtering results based on a value of ≤0.001.

### Whole Blood Epigenetic Analysis

Genomic DNA (gDNA) was extracted from 200 μl whole blood samples using DNeasy Blood & Tissue Kit (Qiagen, Hilden, Germany) following manufacturer’s instructions. Seven hundred fifty nanograms of resulting gDNA samples were subjected to overnight bisulfite conversions with EZ DNA Methylation kit (ZymoResearch, Irvine, CA) in a PCR thermal cycler (16 cycles of 95°C for 30 s, and 50°C for 60 min). To perform global DNA methylation profiling on the Illumina MethylationEPIC beadchips, 160 ng of the bisulfite converted DNA (bcDNA) samples were used. The array procedure started with an overnight whole-genome amplification at 37°C. The amplified products were then enzymatically fragmented, precipitated with isopropanol, then resuspended in hybridization buffer. Following heat denaturation, processed bcDNA samples were hybridized onto MethylationEPIC BeadChips in an overnight incubation at 48°C. The next day, unbound bcDNA were washed off the chips, then single-base extension was performed with the provided DNP-labeled and biotin-labeled dNTPs. After neutralization, labeled extended primers on each array were stained at 32°C in the chamber rack with Cy5 labeled anti-DNP antibodies and Cy3 labeled streptavidin as per manufacturer’s protocol in a 90-min staining procedure. Stained EPIC chips were then sealed, dried, and scanned with the Illumina iScan on a two-color channel to detect Cy3 labeled probes on the green channel and Cy5 labeled probes on the red channel. Using the Illumina GenomeStudio software package, average beta values were calculated by dividing the methylated probe signal intensity by the sum of methylated and unmethylated probe signal intensities. Average beta values range from 0 (completely unmethylated) to 1 (fully methylated) and provided a quantitative readout of relative DNA methylation for each CpG site within the cell population being interrogated. The DNA methylation (DNAm) data were first processed by checking their beta value distributions in R statistical software. The EPIC array beta values were the used to perform hierarchical clustering using either the entire EPIC array or 58 SNP probe’s beta values. The DNAm data quality was assessed by filtering of the EPIC was performed according to this criteria: if probes were interrogated a SNP, had evidence of cross hybridizing to a region of the genome other than the designed target (14), had a SNP present at the CpG target or single base extension of the probe (polymorphic probes) (14). Probes were removed if they had a beadcount <3 in 5% of samples, or had 1% of samples with a bad detection p-value > 0.05. Samples were then normalized using the dasen method (15) bringing the beta value closer together. Correction of blood cell composition was performed to in whole blood samples using linear regression of counts from the complete blood count (16). Batch effects between Sentrix IDs were observed using Principal Component Analysis (PCA) and ComBat (17) was used to correct for for Sentrix ID. Data from technical replicates were also assessed. In order to improve computational efficiency and reduce multiple test correction, 101,864 non-variable CpGs were removed as previously described (18). To analyze the data looking at the effect of vaccination, age or sex in the cohort, a linear mixed effects model was ran with DNAm value as outcome, the other factors as main effects with a covariate of the subject ID as a random effects.

### Proteomic and Lipidomic Analysis

WBC samples were lysed using published methods (19). Proteins were measured using the Pierce™ Bradford Assay and 10 μg per sample was digested as previously described (20). Peptides were desalted using STop-And-Go Extraction tips (STAGE tips) (21), dried using the Vacufuge Plus (Eppendorf) for 45 min, then chemically dimethylated with light, medium, and heavy formaldehyde (22) for triplex analysis of each time point per individual (i.e., two triplex samples were prepared for each individual, with sample from one of the time points spiked into both triplexed samples to use as a reference). After labeling, samples for each triplex were combined, desalted and dried again using STAGE tips and the Vacufuge Plus. Peptides were resuspended in 30 μl of 0.1% formic acid for liquid chromatography and mass spectrometry analysis (LC-MS). Two micrograms per sample was injected into the EasynLC-1000 chromatography system (Thermo) with a 50-cm analytical column, packed in-house with C18, coupled to an Impact II Q-TOF mass spectrometer (Bruker Daltonics, Bremen, Germany), with detailed parameters as previously described (23). Data was analysed using MaxQuant (v1.5.5.1) with default values and “Match Between Runs” activated, searched against the human Uniprot database (downloaded on 15 July, 2017). The mass spectrometry proteomics data have been deposited to the ProteomeXchange Consortium *via* the PRIDE (24) partner repository with the dataset identifier PXD020474.

Plasma lipidomics samples were prepared using a variation of a published protocol (25). Aliquots of 10 μl of plasma were mixed with 180 μl of ammonium bicarbonate (150 mM), followed by extraction with 790 μl of a 7:2 mixture of methyl tert-butyl ether and methanol, respectively. Samples were spiked with 21 μl of internal standard (**Table 4**). Samples were shaken for 15 min at 4°C, and then underwent centrifugation at 3000 × *g* for 5 min, from which 100 μl of the organic portion was taken and dried on a 96-well plate; each was resuspended in 20 μl of ammonium acetate (7.5 mM) in a 1:2:4 mixture of chloroform, methanol, and isopropanol, respectively. The samples were loaded using the TriVersa NanoMate (Advion, Ithaca, NY, USA) infusion robot and a 4-mm infusion chip, and analyzed by the Impact II Q-TOF mass spectrometer (Bruker Daltonics, Bremen, Germany) in positive and negative ionization mode. Raw data were processed using an in-house script. Batch correction was performed on log2-transformed data using ComBat from the “sva” R package (26). A paired *t*-test was used to test for differentially abundant proteins at each post-vaccination time point (Days 1, 3, 7, and 14) compared to the pre-vaccine time point (Day 0). For comparison of non-responders and responders, a *t*-test and multiple-testing correction were applied *via* a custom script in R. For all tests, proteins were filtered for significance using an adjust p-value of 0.05.

**Table 4:** Composition of standards used for analysis of plasma lipidomics.

Lipid name	Quantity in standard
lysophosphatidylglycerol 17:1	50 pmol
lysophosphatic acid 17:0	50 pmol
phosphatidylcholine 17:0/17:0	500 pmol
hexosylceramide 18:1;2/12:0	30 pmol
phosphatidylserine 17:0/17:0	50 pmol
phosphatidylglycerol 17:0/17:0	50 pmol
phosphatidic acid 17:0/17:0	50 pmol
lysophposphatidylinositol	50 pmol
lysophosphatidylserine 17:1	50 pmol
cholesterol D6	1 nmol
diacylglycerol 17:0/17:0	100 pmol
triacylglycerol 17:0/17:0/17:0	50 pmol
ceramide 18:1;2/17:0	50 pmol
sphingomyelin 18:1;2/12:0	200 pmol
lysophosphatidylcholine 12:0	50 pmol
lysophosphatidylethanolamine 17:1	30 pmol
phosphatidylethanolamine 17:0/17:0	50 pmol
cholesterol ester 20:0	100 pmol
phosphatidylinositol 16:0/16:0	50 pmol

### Immune Cell Phenotyping

**Figure 4:**
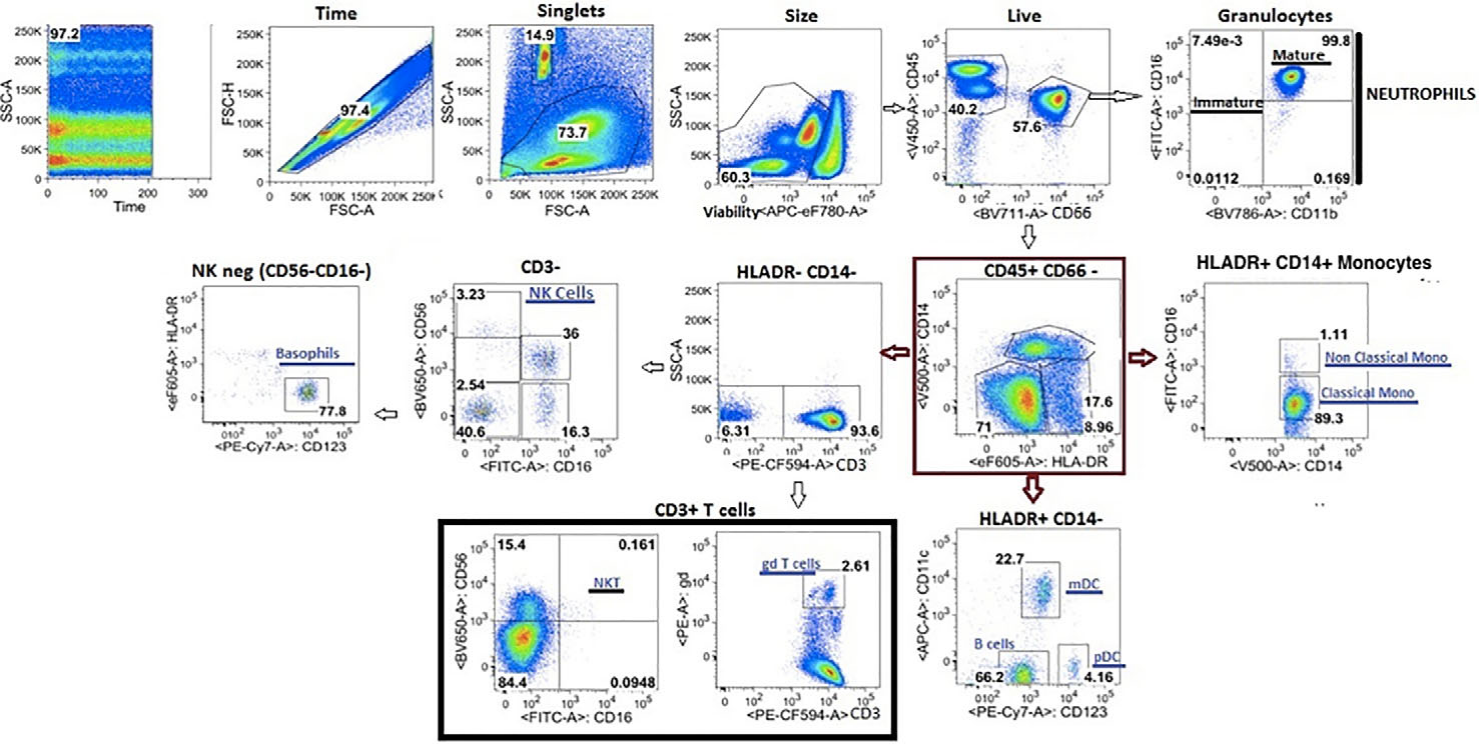
Flow cytometry gating strategy of immune cells in whole blood.

Fixed cells were stained per manufacturer recommendations with different labeled antibodies (**Table 5**) to identify specific cell populations. Flow cytometry data were acquired on a LSRII flow cytometer (BD Biosciences). Flow cytometry raw data were analyzed manually using Flowjo software (version 9.9). In addition, immunophenotyping using automated gating was undertaken on the same samples. This has advantages over manual gating, including increased throughput and identification of specific cell populations with up to 50-dimensional datasets (27). Pre- processing to detect anomalous events obtained during data acquisition was done using the flowCut algorithm (https://rdrr.io/github/jmeskas/flowCut/man/flowCut.htmL). In brief, this approach detects and removes events within time segments for which the fluorescence intensities deviate from the norm. Next, events with either the minimum or maximum value in any of the scatter channels were removed. Singlets were selected using the FSC-A and FCS-W channels. Finally, the data were compensated and transformed using a logical transformation. For each sample, flowDensity (a supervised gating algorithm) (28) was used to determine the thresholds for each marker in each of the biaxial plots in a data-driven manner based on the density distribution of the fluorescence signal. If there were two peaks in the density distribution, the split was located at the minimum between the two peaks. In the case of more than two peaks, the peak-to-peak distance and valley heights was used to determine which split gave the most distinct cut. Otherwise, the algorithm used inflection points. Five challenging populations (live cells, myeloid and plasmacytoid DCs, gamma delta T cells and CD56^bri^CD16^lo^ NK cells) required a second gating step using flowPeaks, an unsupervised clustering algorithm (29). These included populations which were difficult to separate from the background and which could not easily be defined by polygons with sides at pre-defined angles. We defined these cell populations as the union of all of the clusters with centroids inside of the flowDensity-determined boundaries. Cell counts for each of the populations in the gating strategy were obtained and normalized to the bead count, yielding cell counts for a total of 24 immune cell populations (**Figure 4**). Correlation analysis between antibody titres and cell composition were performed. A Spearman correlation was utilized where antibody data were normally distributed and a Wilcoxon rank sum test when antibody data were skewed.

**Table 5:** Antibody panel for single cell immunophenotyping.

Marker	Target	Fluorochrome	Clone	Catalog Number
CD64	Activated leukocytes	Alex 700	10.1	BD #561188
CD11c	Myeloid dendritic cells (mDC)	APC	Bu15	BD #340544
CD123	Plasmacytoid dendritic cells (pDC)	PE-Cy7	6H6	ebio #25-1239-42
CD3	T cell lineage	PE-CF-594	UCHT1	BD #562280
gd TCR	gamma delta T cells (γδ T cells)	PE	B1.1	ebio #12-9959-42
CD56	NK cells	BV650	HCD56	Biolegend #318343
CD11b	Mature and Immature Neutrophils	BV786	ICRF44	Biolegend #301346
CD66	Neutrophils	Biotin/BV711 Streptavidin	ASL-32	Biolegend #92716/BD563262
CD16	Neutrophils, pro-inflammatory and transitional monocytes and NK cell subsets	FITC	3G8	Pharmingen #560996
HLA-DR	Dendritic cells, monocytes and B cells	eFlour605	LN3	ebio #83-9956
CD14	Classical and intermediate monocytes	V500	M5E2	BD #561391
CD45	Pan leukocyte antigen	V450	HI30	BD #560367

### Single Cell Gene Expression

Processing of the frozen 96-well plates containing single cell lysates was performed as previously described (30) with modifications to accommodate an Agilent BioCel automated liquid handling platform (31). Briefly, single cell lysates contained in the 96-well sorted plates were processed in batches of eight plates, with each plate containing wells reserved for 10 pg Universal Human RNA (Clontech) as a positive control, an ERCC-only control (Thermo Fisher), and water as a negative control. cDNA synthesis, lysis, reverse transcription with Smart-seq2, and PCR were carried out in a reduced volume (12.5 µl) and with ERCC internal controls spiked-in at a reduced concentration using a 55 million-fold dilution of the ERCC stock in the first strand cDNA synthesis step. Amplified cDNAs from the eight 96-well plates were consolidated to two 384-well plates and purified with Ampure magnetic particles. A 10-fold diluted portion of each cDNAs was assessed for expression of the Beta-Actin housekeeping gene by qPCR for quality control of the amplified cDNAs.

A cycle threshold (Ct) of ≤35 was used as a cutoff for the selection of 3,072 cDNAs for library preparation and sequencing. A Star liquid handling platform (Hamilton) was used to consolidate cDNAs selected for Illumina Nextera XT library preps into 384-well plates. An automated 1/8th Nextera XT reaction was carried out on 125 pg of the selected cDNAs for the Tn5 tagmentation step, with limited 15-cycle PCR followed by AmPure bead purification. Nextera XT PCR was carried out with a combination of 384 barcode pairs using Nextera barcode sets A and D. Concentrations of the purified Nextera XT reactions were normalized to 1 ng/µl and combined into a 2-ng pool of 384 dual-barcoded samples. RNA-seq was carried out with a total of eight 384 barcoded pools loaded across 16 lanes of an Illumina HiSeq 2500 according to manufacturer’s specifications for a total of 3,072 samples sequenced, including controls. A Hiseq SBS V4 250cycle kit and a Paired End V4 Cluster Kit were used for an estimated 2 million reads per sample.

Single cell RNA-seq data was processed according to published methods (30). Briefly, raw sequencing files were demultiplexing using Illumina barcodes, while sequencing primers and low-quality bases were removed using the Trimmomatic software package (32). Trimmed reads were then aligned using HISAT (33) in two steps: first to a reference of ERCC sequences, and then to GRCh38 (Ensembl). StringTie (33) was used to assemble the resulting alignments into transcript structures and gene expression values (TPM) estimated using GENCODE v25 annotation (Ensembl 87; 10-2016); HTSeq-count (10) was used to generate raw gene alignment counts.

Quality control analysis was performed using sequencing and laboratory metrics, including average Phred score, read complexity, and sample concentration, to classify cell samples as pass or fail using a Random Forest quality control classification model as previously described (34). Expression values for cell samples that passed quality control classification were fed into the Scater and SC3 algorithms for PCA, t-distributed stochastic neighbor embedding (tSNE) visualization, and cluster analysis (35, 36). Unsupervised clustering was performed for the entire dataset, while additional supervised clustering guided by flow cytometry marker panels was performed to investigate within cell type variation. Lastly, cell type marker determination was performed using the SC3 unsupervised clustering results and the NS-Forest algorithm (37). The end result of this computational pipeline produces a set of specimen-specific unbiased cell type clusters, a gene expression matrix with the expression levels of genes in individual single cells grouped into cell type clusters, and a set of sensitive and specific marker genes for each cell type cluster to be used for downstream assays (e.g., quantitative PCR) and semantic representations (38).

### Cell Mediated Immunity

**Figure 5:**
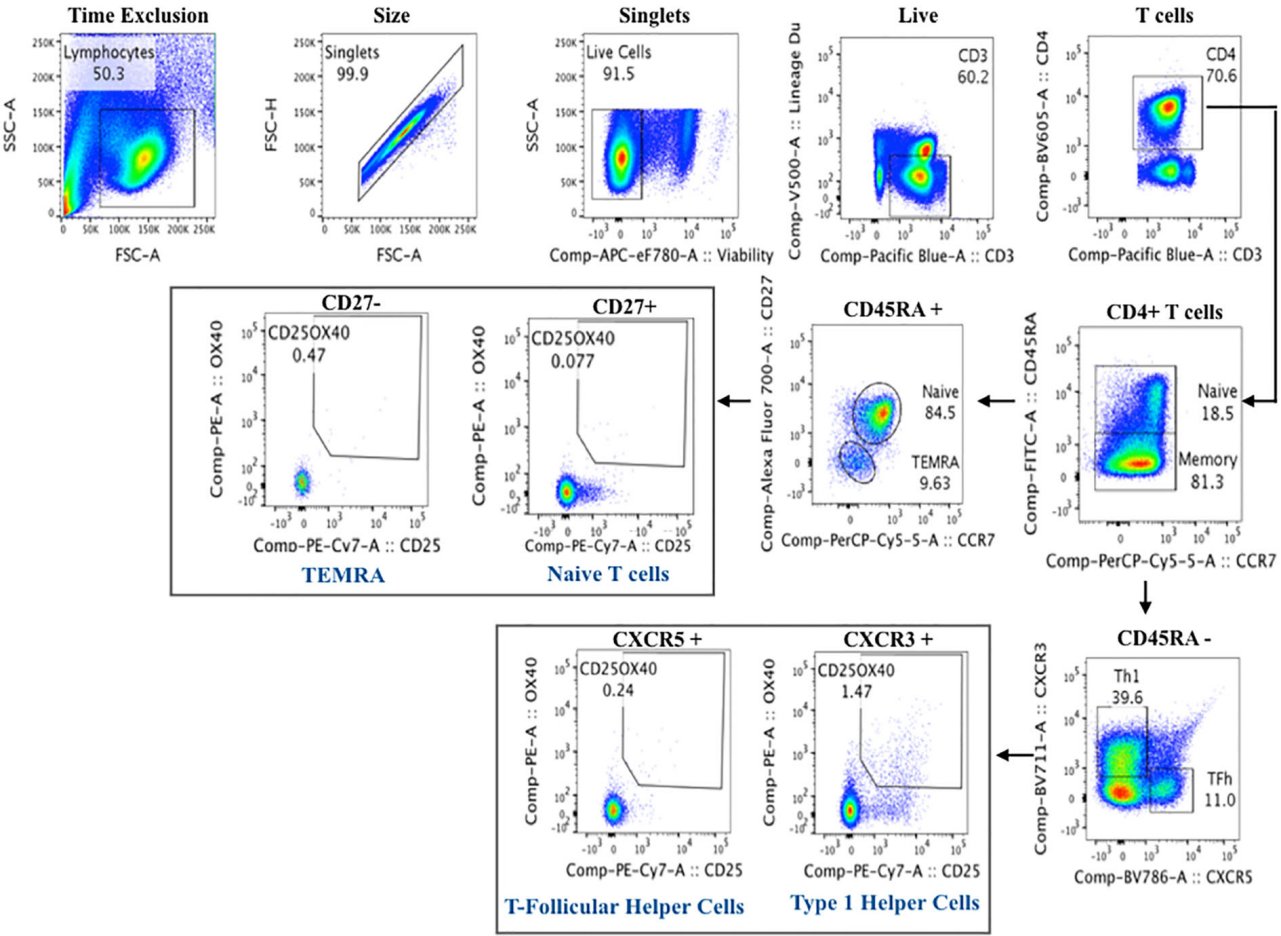
Flow cytometry gating strategy of activated T cells for cell mediated immunity assay to Hepatitis B.

Cryopreserved PBMCs were thawed at 37°C and rested overnight in AIMV serum free medium (MAfisher, MA, USA). Viable cells were counted using an automated cell counter (Nexcelom, Lawrence, MA, USA). Up to 10^6^ PBMCs were plated per well in 96-well plates and either left unstimulated or stimulated with either Staphylococcal enterotoxin B (positive control) or a HB peptide pool (AA labs, San Diego) to assess T cell activation in response to HBV re-stimulation. At 44-h post-stimulation, cells were detached from the wells using 20 μM EDTA (Invitrogen), washed in PBS, and stained with viability dye and antibodies specific to T cell activation markers (**Table 6**). At the end of the incubation, the stained cells were washed and flow data acquired on a LSRII flow cytometer (BD Biosciences). Flow cytomtery data were analyzed using Flowjo software using the gating strategy shown in **Figure 5** (version 9.9, LLC, USA).

**Table 6:** Antibody panel for in vitro T cell mediated immune responses to hepatitis B.

Marker	Target	Fluorochrome	Clone	Catalog number
CD14	Classical and intermediate monocytes	V500	M5E2	BD #561391
CD19	B cells	V500	HIB19	BD # 561121
CD8	CD8 T cells	BV510	RPA-T8	BD # 563256
CD3	T cell lineage	PacBlue	UCHT1	BD # 558117
CD4	CD4 T cells	BV605	SK3	BD #565998
CD45RA	Naive T cells	FITC	HI100	Biolegend # 304106
CCR7	Naive and regulatory T cells	PerCP-Cy5.5	G043H7	Biolegend # 353220
CD27	T and B cell subsets, NK cells	AF700	M-T271	Biolegend # 356416
CD25	Activated T cells	PE-Cy7	M-A251	BD # 557741
OX40 (CD134)	Activated T cells	PE	L106	BD #340420
PDL1 (CD274)	Activated T cells	APC	29E2A3	Biolegend #329708
CXCR5 (CD185)**	T-Follicular Helper Cells	BV785	J252D4	Biolegend #359132
CXCR3 (CD183)**	Type 1 Helper Cells	BV711	G025H7	Biolegend #353732

**Spiked in the cells during incubation and re-stain.

### Response to Innate and Adaptive Immune Stimuli

The TruCulture^®^ systems were developed to provide robust induction of innate and adaptive immune responses (6) and have previously been shown to be more reproducible than conventional PBMC-based stimulations (39). In this context, only pre-vaccine samples were tested with the *Milieu Interieur* (40) platform. RNA was isolated from Trizol whole blood cell samples using the NucleoSpin 96 RNA tissue kit protocol (Macherey-Nagel) with some modifications as previously described^4^. RNA yield, integrity (RIN), and quality (RQS) scores were estimated, quality control validated and pristine samples with an RQS > 4 were processed for gene expression analysis. The NanoString nCounter system was used for the digital counting of transcripts. One hundred nanograms of total RNA were hybridized with the Human Immunology v2 Gene Expression CodeSet according to the manufacturer’s instructions (41). Samples were processed in two batches, within which the samples were randomized. All samples were normalized together following background subtraction of the negative control probes, using positive control probes and housekeeping genes selected by the GNorm method as previously described (42). Quality control for our data involved checking the following metrics: fields of view ≥0.75; binding density 0.05–2.75, linearity of positive controls (R2 ≥ 0.9), and limit of detection for 0.5 fM positive control ≥2 standard deviations above the mean of the negative controls.

Supernatants from TruCulture tubes were thawed on ice and tested by Luminex xMAP technology for a total of 32 proteins including cytokines, chemokines and growth factors as previously described (6). Samples were measured on the Myriad RBM Inc platform (Austen, Texas) according to CLIA guidelines (set forth by the USA Clinical and Laboratory Standards Institute). The least detectable dose for each assay was derived by averaging the values obtained from 200 runs with the matrix diluent and adding 3 standard deviations to the mean. The lower limit of quantification was determined based on the standard curve for each assay and is the lowest concentration of an analyte in a sample that can be reliably detected and at which the total error meets CLIA requirements for laboratory accuracy.

### Anti-HBV Antibody Repertoire Analysis

The repertoire of anti-HBV IgG antibodies was analyzed by isolation of IgG from single-sorted B cells and IgG NGS. For antibody isolation, single IgG+ antigen-specific CD19+ CD20+ B cells were sorted from PBMC samples in 96-well plates using HBV virus-like particles fluorescently labeled with Alexa Fluor-488 and Alexa Fluor-647 dyes. The sort density was one cell per well to enable later antibody chain pairing. Reverse transcription and PCR amplification of heavy and light chain variable genes were performed, and the antibodies were assembled for each source cell. Post-vaccination antibody clonality was compared between different participants. Antibody NGS was undertaken on samples collected before and after the third vaccine dose. For each sample, antibody heavy chains were amplified from mRNA isolated from total PBMCs. Reverse transcription of the RNA was performed using barcoding primers containing unique molecular identifiers (UMIs), as previously described (43). The resulting cDNA was used to amplify antibody heavy chains. Antibody sequence annotation, including VDJ assignment was performed using AbStar (https://github.com/briney/abstar). Error correction of sequencing data was done by UMI-based correction using AbCorrect (https://github.com/briney/abtools/blob/master/docs/source/abcorrect.rst). This combination of AbStar and AbCorrect allows clustering and consensus/centroid generation on just the VDJ region of the antibody sequence in the proper orientation. For clonal lineage assignment, sequences were assigned to clonal lineages using Clonify, as previously described (43). Clonify uses an antibody-specific distance metric, incorporating length-normalized CDR3 edit distance, V and J gene usage, and shared somatic mutations to determine the relatedness of each antibody sequence pair.

### Cytokine Analysis

Single molecule array (Simoa) is an ultra-sensitive single molecule array which is able to detect cytokines at extremely low levels, below the detection methods of conventional immunoassays (44–50). This method integrates conventional bead-based ELISA with microwell-array technology and has limits of detection approaching the attomolar to femtomolar range. Analysis was undertaken for a panel of 15 cytokines (IFN-α, IFN-β, IFN-γ, IFN-ω, IL-1β, IL-2, IL-6, IL-7, IL-8, IL-10, IL-12p40, IL-12p70, IL-15, GM-CSF, and TNF-α), using published methods (48). Briefly, multiplex capture beads were purchased with pre-encoded fluorescent dyes to generate multiple distinct bead populations. Capture antibodies were then coupled onto the paramagnetic beads. Detection antibodies were either purchased with biotin conjugates or biotin conjugated in-house. All reagent solutions (5 × 10^6^ capture antibody-coated paramagnetic beads, biotin-conjugated detection antibodies, 150 pmol/L streptavidin-β-galactosidase reagent, 1× PBS buffer, 100 μmol/L resorufin β-d-galactopyranoside substrate, wash buffers and fluorocarbon oil) were loaded onto the Simoa HD-1 Analyzer (Quanterix, Lexington, MA, USA). Cytokine controls were prepared in 1× PBS containing 25% newborn calf serum. Controls (serial dilutions) and samples fourfold diluted in 1× PBS) were loaded into a 96-well plate and analyzed in triplicate. The samples were screened for 15 cytokines using a total of seven Simoa assays (four 3-plex and three single-plex). Cytokine concentrations were determined *via* hydrolysis of the resorufin β-d-galactopyranoside substrate to generate the fluorescent product resorufin, which was detected *via* excitation/emission of 574 nm/615 nm taken at a 30-s interval, with a threshold of ≥20% increase in fluorescent intensity against the random fluorescent background. Subsequent fluorescent images were taken at excitation/emission wavelengths for the different bead types, as previously described. A four-parameter logistical curve was applied for the average enzymes per bead as a function of cytokine concentration curve fitting.

### Microbiome

Bacterial community composition was analyzed in rectal, skin, oral and nasal samples. The data were generated *via* high-throughput sequencing of bacterial V4-16S rRNA amplicons, using the Illumina MiSeq platform using a refined version of an established analysis pipeline, which has been described previously (51). To identify and remove contaminating sequences, three extraction blanks were included. These were generated by first performing three extraction blanks, then using the eluate from these as a template for PCR amplification, and then sequencing in the same run as the biospecimens. Two positive controls, consisting of cloned SUP05 DNA, were also included (number of copies = 2*10^6). OTUs present in blanks are either introduced from extraction reagents, PCR reagents, or from neighboring samples during either the extraction or the PCR amplification process. Contaminating OTUs were identified as being present in at least 50% of blanks, with count geometric mean plus one standard deviation was greater than the samples, reflecting previously described properties of contaminants arising from extraction or PCR reagents. This approach also avoids removing common contaminating OTUs from neighboring samples either during the extraction or PCR amplification steps, as these are of high relative abundance in the samples but low relative abundance or sporadic presence in the blanks. Contaminating OTUs were removed from the dataset. Samples with total read counts under 1,000 after contaminant removal were also removed from further analysis. The analyses address analytical challenges inherent to microbiome data. First, community composition data are in the form of relative abundance of populations, which do not vary independently, and are not suited to univariate methods. Second, the data are sparse, typically with a large proportion of populations at zero relative abundance in any particular sample. Analysis is robust to sparsity, because rare populations may be functionally important. Third, because whole microbial communities often modulate biological pathways, testing individual populations, which overlooks interactions or correlations among them, may not adequately capture microbiome effects on host responses. To relate microbiome composition to immune responses, multivariate methods were employed, including a workflow for microbiome data analysis in the *mixOmics* *R* package. To maximize the correlated information between multiple datasets and optimally identify the key microbiome features that explain and reliably classify immune phenotypes, multivariate dimension reduction discriminant analysis was used. This method was extended to allow for exploration of non-linear relationships between features, through the use of kernel methods (52) and other approaches. Additional machine learning approaches (e.g., random forests) were used to develop models predictive of anti-HBs antibody

### Lymph Node Cells Analysis

Fluid from LN aspirates was inoculated into ice cold RPMI and transported immediately to the laboratory on ice, before cells were separated by centrifugation at 700 × *g* for 5 min at 4°C. Cells were resuspended in PBS, 2% fetal bovine serum (FBS), counted using crystal violet, and cryopreserved in 80% FBS, 20% DMSO in liquid nitrogen awaiting further analysis. Germinal center (GC) B cells were identified by co-expression of Bcl6 and Ki67, and GC Tfh cells were identified as CXCR5^hi^PD1^hi^ CD4 T cells (4, 53–55). In general, eight (of 31) LN FNA samples were deemed acceptable for analysis using the established criteria of flow cytometry acquisition of >30,000 total cells and >5,000 B cells. In this study, we analyzed all cells present in the CD20+ B cells or CD4 T cell gates

## Conclusions

In this study, we have determined the feasibility of recruiting healthy adults to a study involving multiple blood and tissue sampling, with multiple vaccine doses and 100% retention. Few studies included the immune responses in solid tissues, particularly lymphoid organs with other omics analysis which will certainly lead to a better understanding of the vaccine- induced protective antibody response (56). The choice of bio-sampling time points to perform multi-omic assay was at some degree based on previous vaccine studies; however, a more frequent sampling scheme could be considered for future studies if no budgetary constraints or burden on the participant is not limiting. Laboratory processing of the large number of sample types from such a study is achievable, and data integration tools are available to analyze and interpret the data. Indeed, a subset of the omics analysis performed on the biospecimen collected were integrated and led us to unique insights in regard to adults response to Hepatitis B vaccine (described in “Multi-omic data integration allows baseline immune signatures to predict hepatitis B vaccine response in a small cohort” manuscript). Similar future studies will be needed with different vaccines, in different age groups, and in high-income and low-to-middle-income countries to enable design of future vaccines which are suitable for all populations. As highlighted in this study, large collaborations will be required to ensure the success of these studies.

## Data Availability Statement

The raw data supporting the conclusions of this article will be made available by the authors, without undue reservation.

## Ethics Statement

The studies involving human participants were reviewed and approved by University of British Columbia Clinical Research ethics Board. The patients/participants provided their written informed consent to participate in this study.

## Author Contributions

RB-O, MS, and TK contributed to the design of the study and writing the clinical and lab protocols SM, KM, GB, and MS contributed to the study participant recruitment, biospecimen collection, financial aspects, and clinical data management RB-O, BC, DH, ACL, and NV contributed in processing the biological samples, performed quality control. MH and BS contributed to lymph node aspirate collection from participants. RB-O, BC, ACL and all other authors (not listed above) performed all the multi-omic analysis listed in this method paper. WK was extensively involved in the conceptualization/design of the study, and together with other co-authors in the other aspects from concept through manuscript preparation. All authors contributed to the article and approved the submitted version.

## Funding

DD acknowledges support from the Laboratoire d’Excellence Milieu Intérieur Consortium (grant no. ANR-10-LABX-69-01). MS is supported *via* salary awards from the BC Children’s Hospital Foundation, the Canadian Child Health Clinician Scientist Program, and the Michael Smith Foundation for Health Research. This work was funded by the Human Vaccines Project.

## Conflict of Interest

MS has been an investigator on projects funded by GlaxoSmithKline, Symvivo, Sanofi Pasteur, Seqirus, Pfizer, Merck and VBI Vaccines. All funds have been paid to his institution and he has received no personal payments. DW is a board member and equity holder of Quanterix Corporation.

The remaining authors declare that the research was conducted in the absence of any commercial or financial relationships that could be construed as a potential conflict of interest.

## Abbreviations

Anti HBs, Anti Hepatitis B surface antigen; bcDNA, Bisulfate converted DNA; cDNA, Complementary Deoxyribonucleic Acid; CMV, cytomegalovirus; DC, Dendritic Cells; DE, Differentially Expressed; DIABLO, Data Integration Analysis for Biomarker discovery using Latent component method for Omics studies; DNAm, DNA Methylation; dNTP, deoxynucleotide Triphosphate; ELISA, enzyme Linked Immunosorbent Assay; ERCC, External RNA Controls Consortium; FSC-A, Forward SCatter Area; gDNA, genomic DNA; HBV, Hepatitis B Vaccine; HIV, Human Immunodeficiency Virus; IgG, Immunoglobin G; IU, International Units; LN, Lymph Node; mRNA, Messenger Ribonucleic Acid; NGS, Next-Generation Sequencing; NK, Natural Killer; PBMC, Peripheral Blood Mononuclear Cells; PBS, Phosphate Buffered Saline; PCA, Principal Component Analysis; PCR, Polymerase Chain Reaction; PPI, Protein Protein Interactions; RNA, Ribonucleic Acid; RNAseq, Ribonucleic Acid Sequencing; SEB, Staphylococcal enterotoxin B; SiMoA, single Molecular Array; SNP, Single Nucleotide Polymorphism; SSC-A, Side SCatter Area; UMI, Inique Molecular Identifiers; VDJ, Variable Diversity and Joining genes; VEC, Vaccine Evaluation Center; WBC, White Blood Cells.
